# Are females more variable than males in gene expression? Meta-analysis of microarray datasets

**DOI:** 10.1186/s13293-015-0036-8

**Published:** 2015-10-29

**Authors:** Yuichiro Itoh, Arthur P. Arnold

**Affiliations:** Department of Integrative Biology & Physiology, Laboratory of Neuroendocrinology of the Brain Research Institute, University of California, 610 Charles E. Young Drive South, Los Angeles, CA 90095-7239 USA

**Keywords:** Sexual differentiation, Sex bias, Gene expression

## Abstract

**Background:**

The majority of preclinical biomedical research involves studies of males rather than females. It is thought that researchers have avoided females based on the idea that female traits are more variable than those of males because of cyclic variation in effects of ovarian hormones.

**Methods:**

To test the assumption of inherently greater female variability, we analyzed 293 microarray datasets measuring gene expression in various tissues of mice and humans, comprising analysis of more than 5 million probes.

**Results:**

Meta-analysis showed that on average, male gene expression is slightly more variable than that of females although the difference is small. We also tested if the X chromosome of humans shows greater variability in gene expression in males than in females, as might be expected because of hemizygous exposure of polymorphic X alleles but again found little sex difference.

**Conclusion:**

Our analysis supports and extends previous studies reporting no overall greater phenotypic variability in females.

**Electronic supplementary material:**

The online version of this article (doi:10.1186/s13293-015-0036-8) contains supplementary material, which is available to authorized users.

## Background

Recent analysis of published articles indicates that in numerous biomedical fields, male animals are used as subjects more than females [1–4]. Moreover, many studies fail to report the sex of animals, tissues, or cells used in the study so that it is impossible to assess whether the sex of the animal or tissue is an important variable. The male bias raises the concern that scientific findings may be applied with greater certainty to males than females.

Researchers may avoid studying female rodents because they wish to avoid the variability thought to be caused by the estrous cycle. The estrous cycle of female mice is about 4–5 days in length and involves changes in levels of estradiol, progesterone, gonadotrophins, and gonadotrophin releasing hormone [5]. These hormones can have potent effects on gene expression and other phenotypes, including epigenetic changes across the genome [6–8]. On the other hand, group-housed male mice establish a dominance hierarchy leading to individual differences in the level of testosterone, which would also be expected to increase variation in phenotype [9]. Moreover, social status of mice could influence levels of glucocorticoids.

Prendergast et al. [10] analyzed 293 articles to compare the variability of phenotypes between gonad-intact male and female mice. They found that females are not more variable than males and that under some conditions males are more variable than females. The amount of male variability was reduced in mice housed as individuals, supporting the idea that phenotypic variability in males might be related to social factors, glucocorticoids, and/or testosterone levels.

It has been postulated that in outbred populations such as humans, X genes may have more variable effects on phenotype in males than in females because the effect of each X gene variant in females is averaged with the effect of the X allele on the other X chromosome, whereas the X chromosome variation is not reduced by averaging in males because of hemizygous exposure of the X chromosome. On the other hand, males experience only the maternal imprint on X alleles, whereas females experience the imprints of both parents, which could increase variability of X gene expression in females relative to males.

The development of microarrays revolutionized the global analysis of gene expression in diverse tissues and has often been used to detect patterns of expression across the genome. Microarrays and other high-throughput methods are sensitive, accurate, and suitable for the assessment of effects of sex, genotype, environment, and treatment variables on global patterns of gene expression.

Here, we performed a meta-analysis of online databases of gene expression based on microarray analysis, to compare phenotypic variation in males and females. We analyzed data from human and mice. We looked for evidence that gene expression is more variable in females than in males, either globally throughout the genome or in a subset of genes that would be large enough to be observed as a shift in the distribution of expression variance relative to males. Based on the analysis of more than 5 million probes, we found that variation of gene expression was quite similar between males and females, with slight overall bias towards greater variability in males. We also compared variation in expression of X and autosomal genes in humans and mice and found little evidence for greater variability of expression of X alleles relative to autosomal alleles in both species.

## Methods

### Microarray data analysis

We selected for analysis a total of 293 datasets (103 for human, 190 for mouse) obtained from the GEO database (http://www.ncbi.nlm.nih.gov), which compared male and female human or mouse samples (Additional file 1: Table S1). These datasets report gene expression levels in a variety of tissues (Additional file 2: Figure S1) based on microarray expression profiling using a variety of platforms. The goal was to obtain as much data as possible, to avoid any bias that might be specific to an individual microarray methodological approach. Datasets were included if the study compared males and females, comprised at least three independent samples per sex, and if any treatment or disease condition was applied equally to both sexes (Additional file 1: Table S1). We attempted to analyze all datasets that met these criteria. In a subset of datasets (87 for human, 190 for mouse), we were able to identify probes for genes that are encoded on the X chromosome or autosomes. We analyzed patterns of variation of X and autosomal genes in both sexes. Data from mice came from studies in which the estrous cycle of mice was not monitored by the investigators.

Statistical analyses and production of graphs were performed in the statistical environment R [11]. The filtered probes were quantile normalized using the “affy” package from Bioconductor (http://www.bioconductor.org/). The variability of gene expression was measured by coefficient of variation (CV, standard deviation divided by the mean). We compared CV of the two sexes within each dataset. The CV is meant to allow comparison of variation in data with different means because CV compensates for the increase in variation as the mean increases.

## Results

**Fig. 1:**
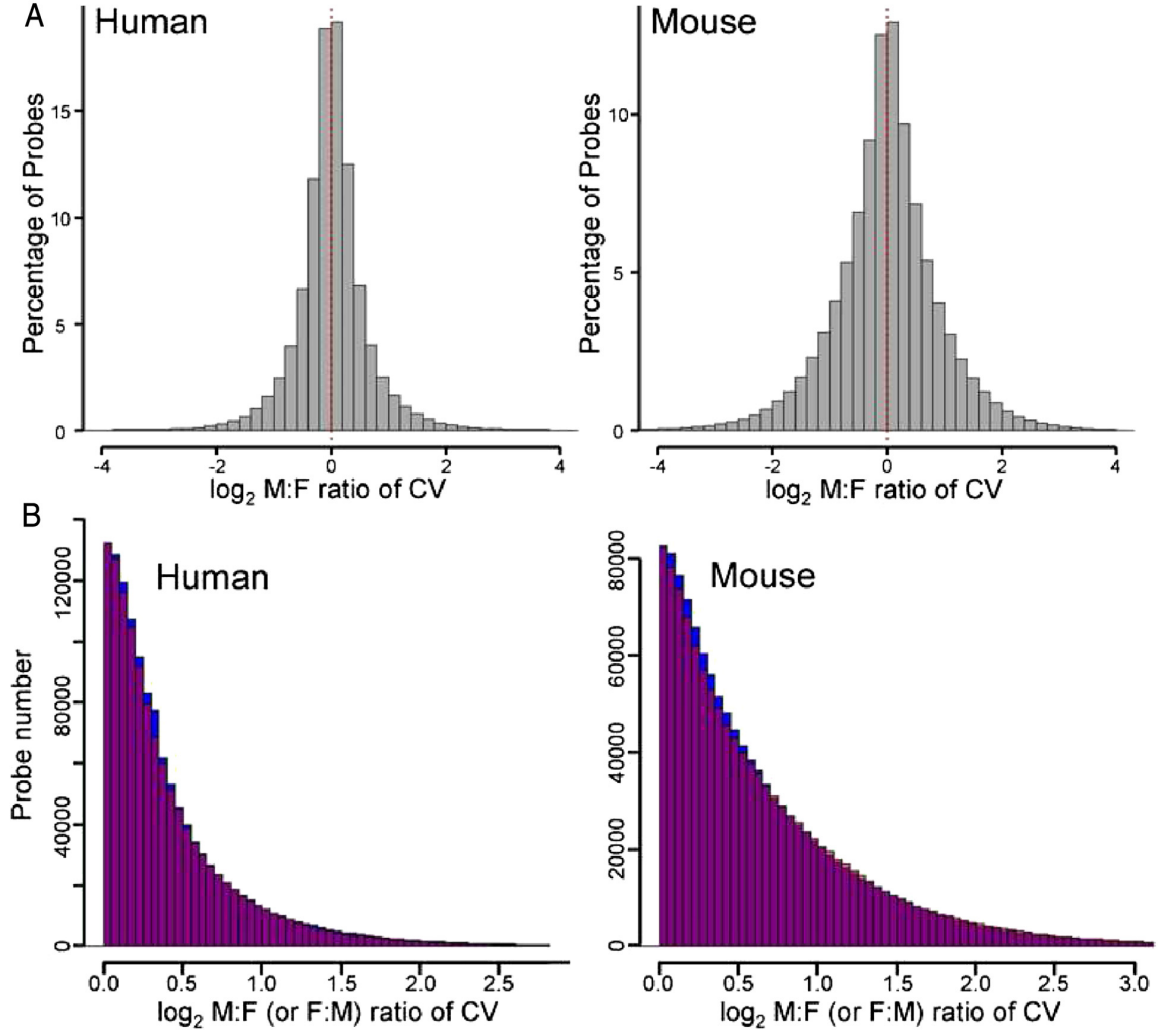
Large scale analysis of male to female ratios of coefficient of variation (CV). **a** Histograms of log_2_ transformed male-to-female ratios of CV. There were 2,665,771 probes for human, 2,426,681 probes for mouse. **b** Histogram comparing the number of probes for which CV was higher in males (*blue*; M > F) or in females (*red*; F > M) at each ratio bin. The region of overlap of blue and red bars is shown as *purple*. At most bins of CV ratios, slightly more probes showed M > F CV

We first calculated the CV for every microarray probe within each sex from 293 datasets from human or mouse, totaling 5,092,452 probes. The male-to-female ratios of those CV values were log_2_ transformed and graphed as a histogram for the human and mouse (Fig. 1a). The histogram is centered around sexual equivalence (a log_2_ ratio of 0, M:F ratio of 1) but with slightly more log_2_ ratios above 0 (slight male bias) in both species. The minor male bias is shown in a graph comparing the number of probes that have the same degree of bias in males or females (Fig. 1b), where many ratio bins had slightly higher number in males than females. If we can assume that the expression of each probe in each study is a statistically independent event, the sex difference in histograms in Fig. 1b was statistically significant (Kruskal-Wallis rank sum test: *p* value <2.2e-16 for mouse, *p* value = 4.362e–12 for human; Wilcoxon rank sum test: *p* value <2.2e–16 for both). We also tested if the filtration threshold influences the pattern of histogram in Fig. 1. For this analysis, we chose microarray datasets with the same platform (human Affymetrix), where filtration thresholds would be more comparable. Genes were filtered out if their expression was below thresholds of 100, 200, 500, or 1000. With all filtration thresholds, slight male biased pattern was consistent in all histograms (Additional file 3: Figure S2).

In this study, microarray datasets from brain contribute disproportionately to the data of Fig. 1, especially in humans (Additional file 2: Figure S1). Therefore, we asked if the pattern in Fig. 1 reflects mostly brain and whether sex bias in variation might differ in non-brain tissues. Additional file 4: Figure S3 separates the analysis for brain and non-brain tissues in human and mice. All of these analyses show either very slight male bias as in Fig. 1, or sexual equivalence of CV, and therefore do not support the idea that degree of sexual bias in CV differs significantly in brain relative to other tissues.

**Fig. 2:**
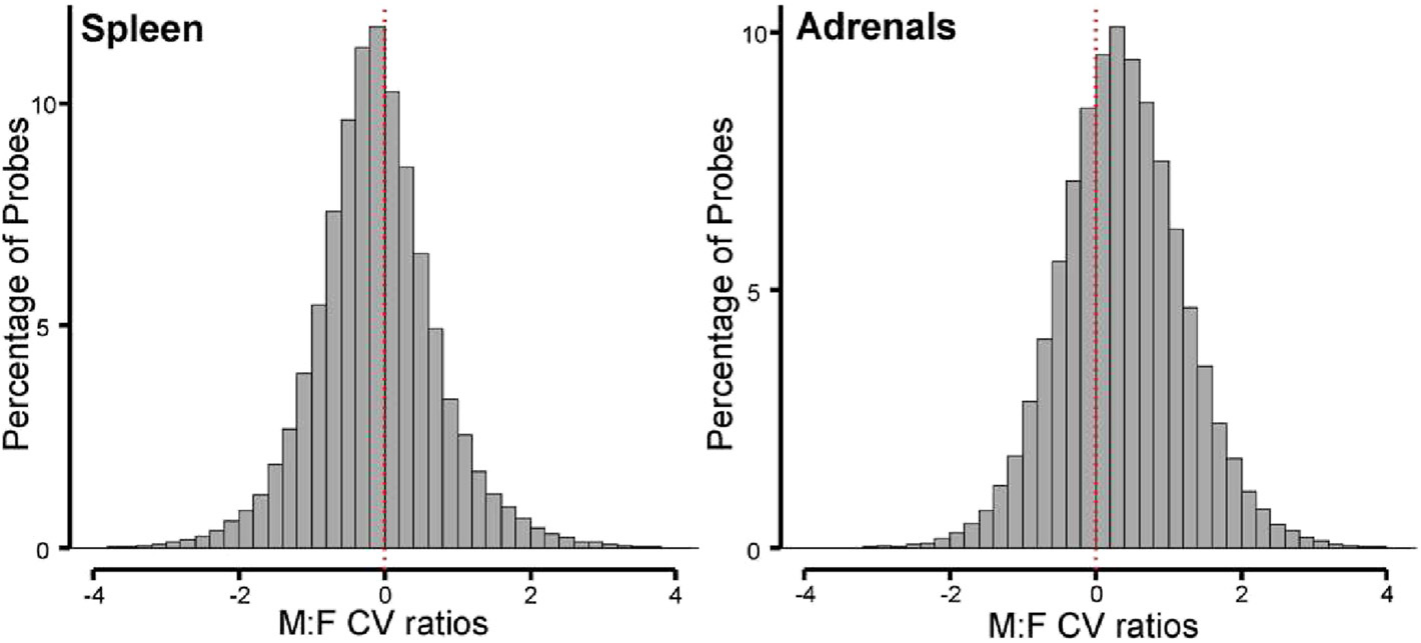
Tissue-specific bias in the variability of gene expression in a restricted set of microarray studies [12]. In mouse spleen, more probes show higher CV in females than in males. In mouse adrenals, more probes showed higher CV in males than females

We analyzed datasets from specific tissues to assess if the overall sexual balance of CV ratios in Fig. 1 was because of tissue-specific sex differences that cancel each other out when considering all tissues combined. For this analysis we selected datasets from one laboratory using similar methods across tissues (GSE9904, GSE9907, GSE9908, GSE9895) [12]. In expression data from spleen, female mice had higher variation in gene expression, but from adrenals, males had higher variability (Fig. 2, Table 1). The kidney and muscle also showed higher variability in male than female mice (Table 1).

**Table 1:** Tissue-specific sex bias of gene expression variation. Four mouse tissues were separately analyzed as examples, and histograms for spleen and adrenals were shown in Fig. 2 (GSE9904, GSE9907, GSE9908, GSE9895: [12])

Tissue	Number of datasets	Number of probes	Male CV		Female CV		*p* value for sex
			Mean	SD	Mean	SD	
Adrenals	8	67,544	0.433	0.273	0.331	0.204	<2.2e–16
Kidney	8	67,544	0.419	0.257	0.372	0.219	<2.2e–16
Skeletal muscle	8	67,544	0.316	0.185	0.286	0.172	<2.2e–16
Spleen	8	67,544	0.388	0.221	0.418	0.229	<2.2e–16

**Fig. 3:**
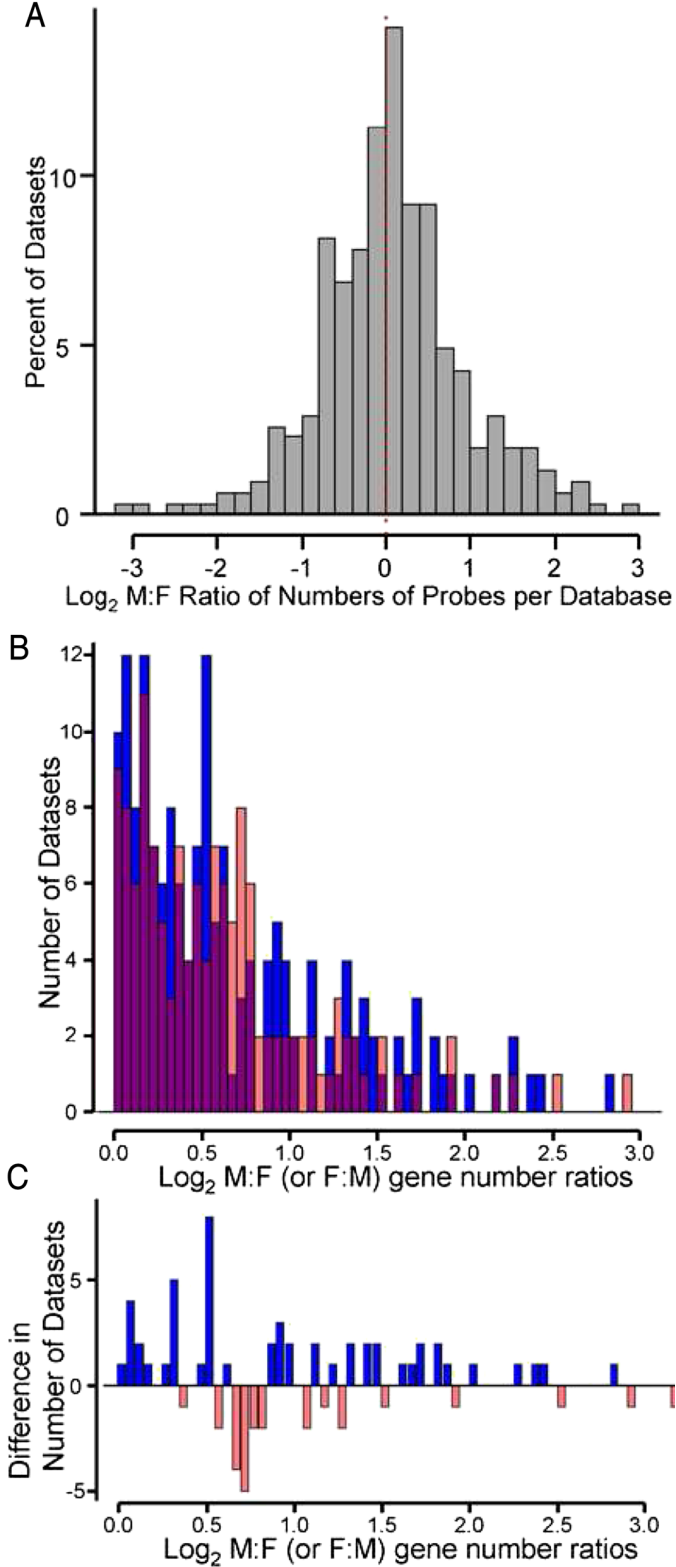
Analysis of sex differences in CV, dataset by dataset. In each dataset, the number of genes with male bias in CV was divided by the number with female bias in CV, and then the M:F ratio was log_2_ transformed. **a** The histogram shows the distribution ratios of 293 datasets. Modal ratios were near sexual equality (log_2_ ratio of 0). **b** Datasets showing more probes with higher CV in males than females are shown in *blue*, and numbers of datasets with more probes with higher CV in females than males are shown in *red*. Overlap of blue and red bars is shown as *purple*. The difference in male and female histograms was not statistically significant. **c** The difference in numbers of datasets at each CV ratio. *Up* shows higher CV in males than females; *down* shows higher CV in females than males

We next analyzed the number of datasets that show sex bias. In some datasets, the numbers of probes showing greater variability in one sex was greater than the number showing greater variability in the other sex. Figure 3a shows the distribution of datasets as the ratio within each study of the number of probes with higher CV in males, divided by the number of probes with CV higher in females. This analysis shows that in some cases, the ratios of number of probes can be quite biased in one direction or the other, with a small number of studies showing as much as 8-fold greater numbers of probes (absolute log_2_ ratios as large as 3) showing greater CV in one sex compared with the other. Nevertheless, the log_2_ modal ratio in Fig. 3a is close to 0 (sexual equality in numbers of probes showing higher CV in each sex), with a slight shift in the distribution towards greater ratios in males, reminiscent of Fig. 1a. The male bias in CV is illustrated further by comparing the amount of sexual bias bin-by-bin (Fig. 3b, c). The shift towards greater variability in males by these analyses was not statistically significant (for Fig. 3b, Kruskal-Wallis rank sum test and Wilcoxon rank sum test, both *p* = 0.91).

**Fig. 4:**
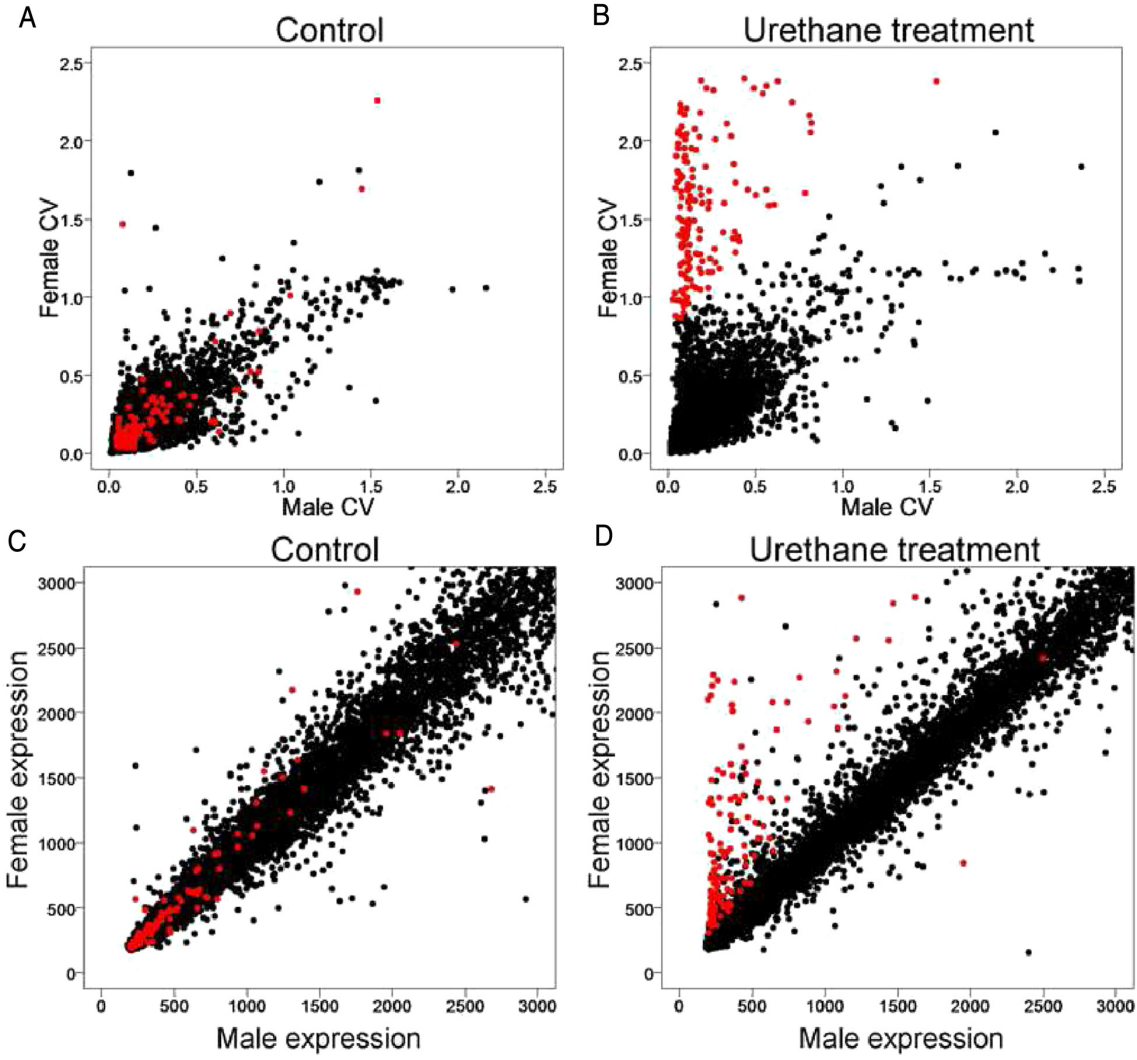
Urethane treatment influences a female bias of CV. **a**, **b** Male and female gene expression CVs were plotted for saline (**a**) or urethane treated (**b**) mouse lung samples. Genes with CV higher in females than male in urethane treatment group (**b** female CV—male CV > 0.8) are defined and shown in *red* in **b**, and the same genes are plotted in *red* in all other figure panels. (**c**, **d**) A group of genes is influenced by urethane treatment in sexually biased manner. Male and female average expression values are plotted as scatter plot. *Red filled circles* are the female higher CV genes categorized in **b**. The data are from GEO (accession: GSE16510, [13])

In some datasets, potentially interesting sex-specific patterns of treatment on variation emerged. For example, in mouse lung studied by Franco et al. [13], the CV of expression was well correlated in males and females across probes in control mice (Fig. 4a). However, treatment with urethane increased the expression CV of a subset of probes in female only (Fig. 4b). The female-specific increase in CV is related to an overall urethane-induced increase in the level of expression of these genes in females relative to males (Fig. 4c, d). Because the CV metric adjusts for the general correlation between mean and variance, this analysis suggests that for the red genes in Fig. 4, urethane caused a female-specific increase in variance of expression that is not accounted for simply by the increase in level of expression.

**Fig. 5:**
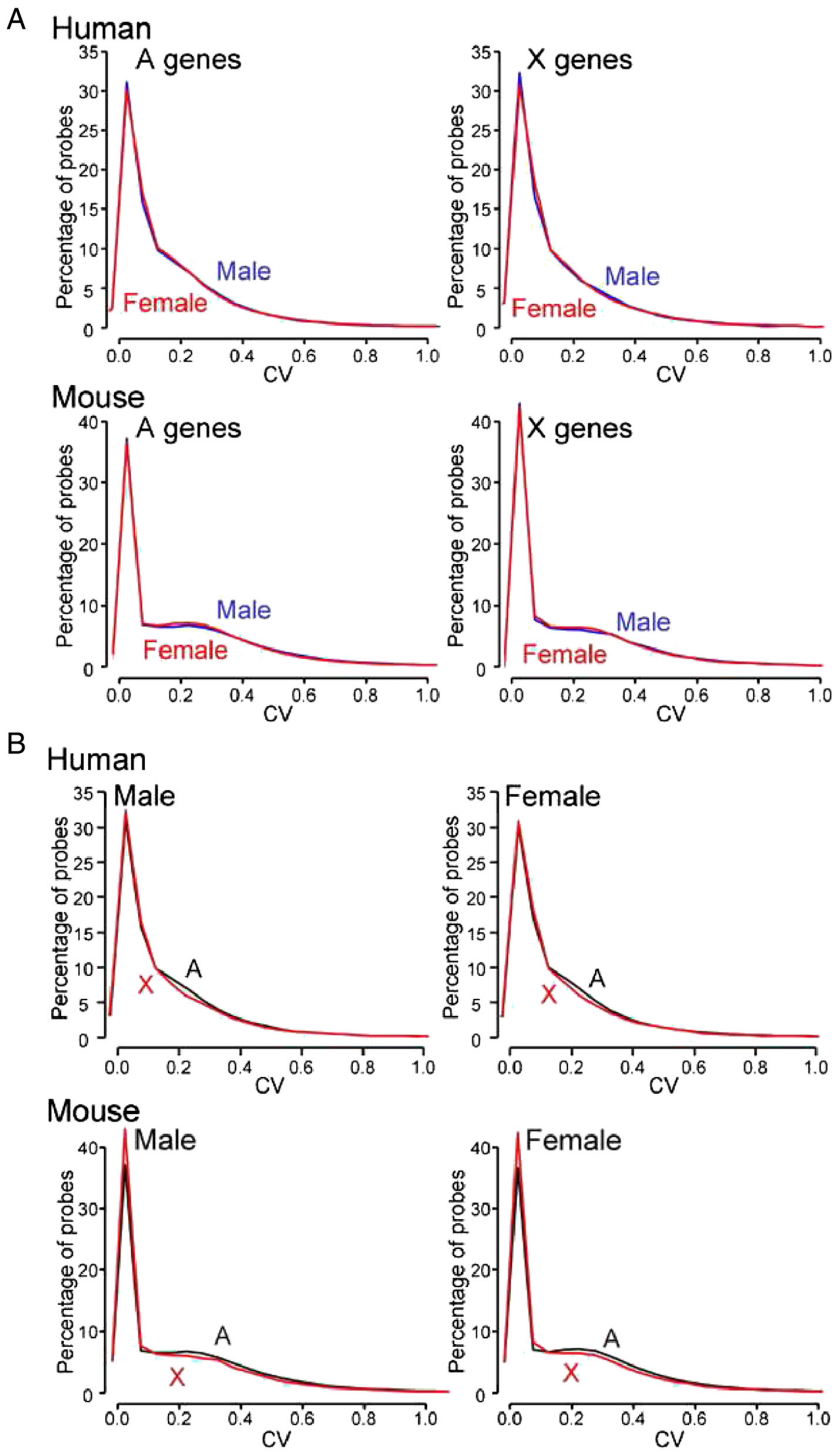
Comparison of CVs between X and autosomal (A) genes in both sexes. **a** The percentages of male or female probes with specific CV values are graphed for CV values less than 1. **b** The curves from **a** are regraphed to allow direct comparison of A and X genes for male and female separately, to illustrate the similarity of the two sexes in both populations. In both human and mouse, the CVs for X genes are similar to those for A genes except for minor differences in the CV range 0.2–0.4. A total of 87 human and 190 mouse datasets were used in this analysis

The hemizygous exposure of X alleles in males is predicted to increase variability of expression of X genes of males relative to females, in outbred populations. We sought evidence of this effect by comparing the CV of male and female in autosomal and X genes (Fig. 5a). We anticipated that this effect might be observed more in samples from humans, which are genetically heterogeneous, whereas it might be absent in data from laboratory mice, which are often inbred or have a restricted range of environments and therefore might show little difference within-study of variability in expression of X genes. The graphs for female were quite similar to those of males, in both autosomal and X genes (Fig. 5a). We also compared CV of autosomal and X genes within sex and found little difference (Fig. 5b). A small difference occurred at CVs in the range 0.2–0.4, whereby autosomal genes had slightly greater CV than X genes in both sexes. Although this small difference was statistically significant (Kruskal-Wallis rank sum test and Wilcoxon rank sum test: *p* value <2.2e–16) because of the large number of probes analyzed, it is not likely to be biologically meaningful. Finally, we focused on the genes with high CV, based on the idea that the sex difference in CV of X genes might be more salient among those genes with the greatest CV. In humans, X genes with greater CV values were found more often in males than in females (Table 2). For mouse datasets, the probe numbers with high CV were too small to conduct a similar analysis.

**Table 2:** Groups of genes from 87 human datasets were categorized according to those that exceeded progressively large CV cutoff values. The percentage of genes in each group that are X-linked is shown in the table, whereas the percentage that was autosomal is 100 minus the number shown. Data for mice is not shown because not many genes showed higher CV values

	Human	
	Male	Female
CV > 1	5.03	4.82
CV > 2	5.23	4.91
CV > 3	4.87	4.54
CV > 4	4.51	4.68
CV > 5	6.91	3.70
CV > 6	7.51	3.44
CV > 7	8.59	3.33

## Discussion

We analyzed 293 microarray gene expression datasets utilizing more than 5 million probes to address the issue of gene expression variability in male and female humans and mice. We found that the variability of gene expression, measured by CV, was similar in the two sexes. The result provides no support for the hypothesis that female mice or humans are generally more variable in phenotype because of their estrous or menstrual cycles or other variables. Indeed, males were on average slightly more variable in some measures of gene expression. Although one sex was sometimes more variable in overall gene expression in specific tissues, the sex bias was in either direction, depending on the tissue, suggesting that sources of sex-specific variability might differentially affect specific tissues. Our results confirm and extend those of previous studies [3, 10] that found no evidence for generally greater variability of various phenotypes in gonad-intact mice, when, as in the present study, estrous stage was not monitored in females. We found that studying sex differences in gene expression can provide an interesting perspective to separate specific populations of genes for further analysis, for example in the study of Franco et al. [13], which provided data that urethane causes a female-specific increase in variability of a selected population of genes (Fig. 4). Finally, our results provide little evidence that X-linked gene expression in humans was more variable in males than in females, as might be expected because of the male’s hemizygous X chromosome.

Sex differences in any phenotype are caused by two kinds of mechanisms, ontogenetic (factors that are inherently different between each male and female, which cause sexual differentiation of tissues), and population-level mechanisms (factors that act in a greater proportion of individuals of one sex than the other, leading to average differences among males and females). Ontogenetic factors include the effects of gonadal hormones, both organizational (permanent, differentiating) and activational (reversible) effects [14]. Variation in the effects of gonadal hormones would be expected to produce the largest sex differences in variability of traits, and accordingly, it is temporal or socially induced variation in effects of gonadal hormones that is most often suggested as a source of sex differences in trait variance. The population-level factors include the following: (1) Hemizygous exposure of X alleles. Variation in X alleles induces more variation in phenotypes of males than females because the effect of each variant is averaged across two alleles in females but is expressed fully in individual males. For example, Fragile X syndrome and other types of X-linked mental retardation affect human males more than females [15, 16]. The averaging process should reduce variability among females relative to males. (2) Individual sexually antagonistic autosomal alleles (conferring different fitness effects in males and females) may occur more in one sex than the other because sex-specific deleterious or lethal alleles might drop from the population of one sex more than the other. (3) “Mother’s curse” [17, 18]: because the mitochondrial genome is passed from mother to daughter, male-disadvantageous alleles may build up if they confer advantages to females and disproportionately promote disease in males.

Although sex differences in level of gene expression are normally thought to contribute to sex differences in physiology or disease, sex-biased variation itself, caused by any type of ontogenetic or population-level effect, can cause one sex to reach a threshold for disease or lethality more than the other sex.

The population-level sources of sex-biasing factors will operate only in genetically heterogeneous populations, not in inbred strains. Among the samples analyzed here, therefore, we expected that the human datasets would represent measures of genetically heterogenous individuals, whereas the mouse datasets would often come from inbred lines. This species difference may have contributed to the greater CV of probes in datasets from humans than from mice (Fig. 5). Some evidence suggests that X genes showing high CV values were more likely to occur in males than females (Table 2). Otherwise, we found little evidence that X genes had greater variability in males than females in humans (where it might have occurred) than in mice (where we did not expect it). Because X genes drive and are driven by autosomal genes within gene networks, it is likely that any tendency for greater variation in X gene expression in males is blunted by network feedback or other interactions with autosomal genes, which comprise the vast majority of interacting partners of X genes within gene networks and which would not be expected to show an overall inherent sex bias.

## Conclusion

Based on extensive analysis of microarray datasets measuring gene expression in both sexes of mice and humans, we found no evidence that variability of gene expression is generally greater in females than males.

## Additional files

Additional file 1: Table S1. A list of microarray databases analyzed in the present study. Sample number indicates the number of independent replicate tissues or animals. Additional file 2: Figure S1. Distribution of tissues measured by datasets in this study. Additional file 3: Figure S2. Histograms of log_2_ transformed male to female ratios of coefficient of variation (CV) with four different filtration thresholds. Thirty human Affymetrix microarray data were selected for this analysis. The distribution of CV is slightly male higher, and this pattern is consistent regardless of the threshold of filtration. Additional file 4: Figure S3. Histograms of log_2_ transformed male to female ratios of coefficient of variation (CV) for brain and non-brain tissues.
