# Supplementary figures and images for: Are females more variable than males in gene expression? Meta-analysis of microarray datasets
Source: Biol Sex Differ. 2015 Oct 29;6:18. doi: 10.1186/s13293-015-0036-8 (PMC4640155; doi:10.1186/s13293-015-0036-8)

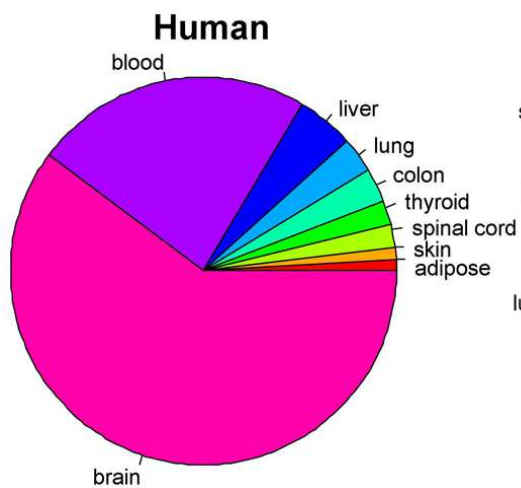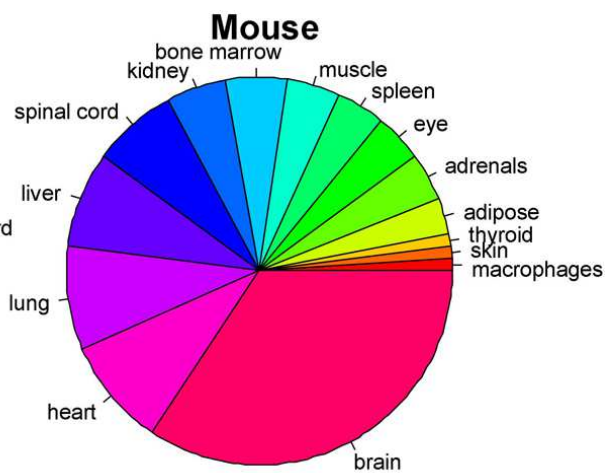

Supplement: Additional file 2: Figure S1. — Distribution of tissues measured by datasets in this study. [file 13293_2015_36_MOESM2_ESM.pdf]

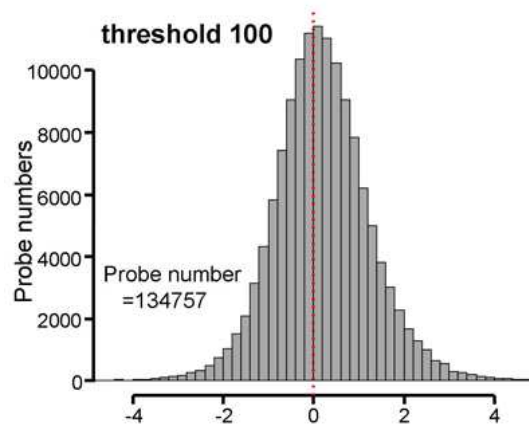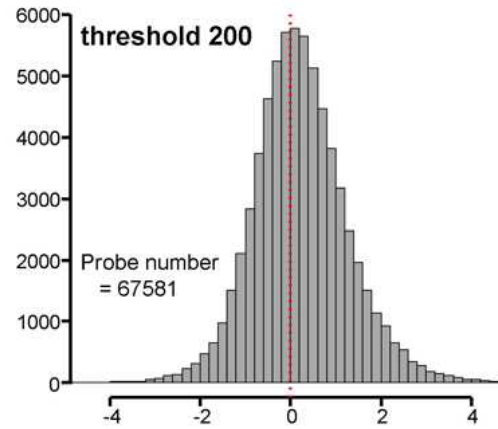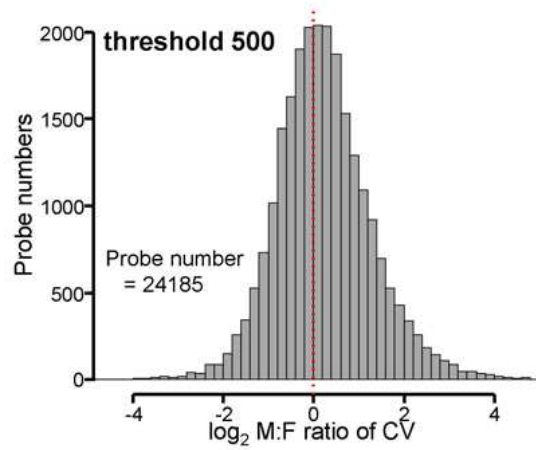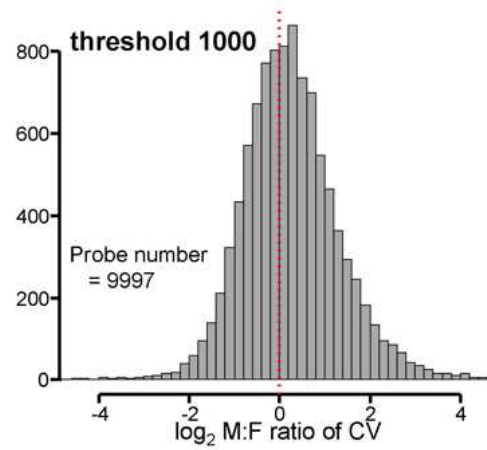

Supplement: Additional file 3: Figure S2. — Histograms of log2 transformed male to female ratios of coefficient of variation (CV) with four different filtration thresholds. Thirty human Affymetrix microarray data were selected for this analysis. The distribution of CV is slightly male higher, and this pattern is consistent regardless of the threshold of filtration. [file 13293_2015_36_MOESM3_ESM.pdf]

## Human

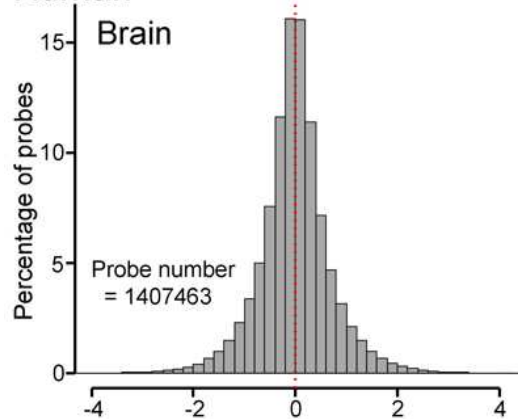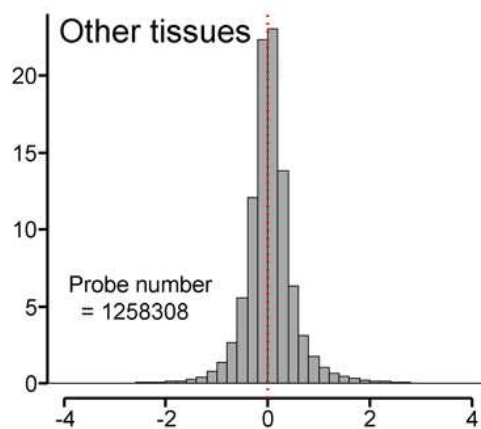

## Mouse

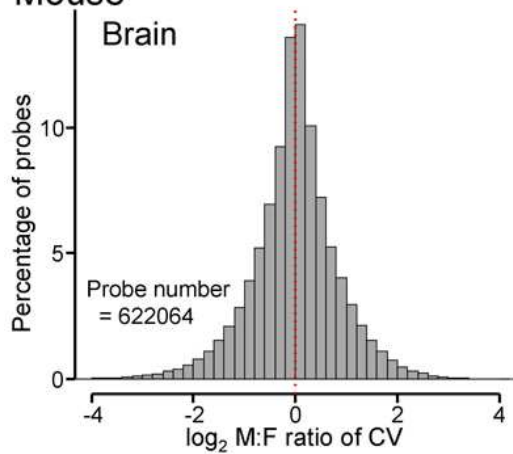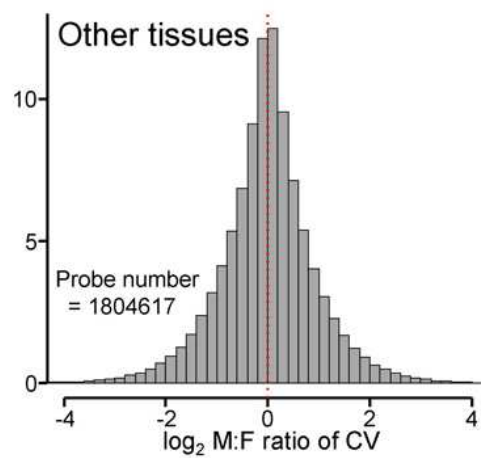

Supplement: Additional file 4: Figure S3. — Histograms of log2 transformed male to female ratios of coefficient of variation (CV) for brain and non-brain tissues. [file 13293_2015_36_MOESM4_ESM.pdf]
